# Influence of Lateralization and Distalization on Joint Function after Primary Reverse Total Shoulder Arthroplasty

**DOI:** 10.3390/bioengineering10121409

**Published:** 2023-12-11

**Authors:** Umile Giuseppe Longo, Edoardo Franceschetti, Arianna Carnevale, Emiliano Schena, Giulia Cozza, Giovanni Perricone, Marco Edoardo Cardinale, Rocco Papalia

**Affiliations:** 1 Fondazione Policlinico Universitario Campus Bio-Medico, Via Álvaro del Portillo, 200, 00128 Rome, Italy; e.franceschetti@policlinicocampus.it (E.F.); arianna.carnevale@policlinicocampus.it (A.C.); e.schena@unicampus.it (E.S.); giulia.cozza@live.it (G.C.); giovanni.perricone@unicampus.it (G.P.); marco.cardinale@unicampus.it (M.E.C.); r.papalia@policlinicocampus.it (R.P.); 2 Research Unit of Orthopaedic and Trauma Surgery, Department of Medicine and Surgery, Università Campus Bio-Medico di Roma, Via Álvaro del Portillo, 21, 00128 Rome, Italy; 3 Laboratory of Measurement and Biomedical Instrumentation, Department of Engineering, Università Campus Bio-Medico di Roma, Via Álvaro del Portillo, 21, 00128 Rome, Italy

**Keywords:** reverse shoulder arthroplasty, shoulder, biomechanics, lateralization, distalization, DSA, LSA, clinical outcomes, kinematic outcomes

## Abstract

The purpose of this study was to investigate how lateralization shoulder angle (LSA) and distalization shoulder angle (DSA) are related to clinical and kinematic outcomes after reverse total shoulder arthroplasty. Thirty-three patients were evaluated at least six months postoperatively. The Single Assessment Numeric Evaluation (SANE), Constant Murley Score (CMS), Simple Shoulder Test (SST), and Visual Analogue Scale (VAS) were used. Shoulder kinematics was evaluated with a stereophotogrammetric system. LSA and DSA inter-rater reliability was analysed through the interclass correlation coefficient (ICC). Stepwise forward linear regression analysis was conducted between LSA and DSA with clinical scales and kinematic measures, between which a correlation analysis was conducted. The inter-rater reliability for LSA (mean ICC = 0.93) and DSA (mean ICC = 0.97) results were good to excellent. Greater LSA values were associated with higher peaks of internal rotation (*p* = 0.012, R^2^ = 0.188) and range of motion (ROM) (*p* = 0.037, R^2^ = 0.133). SANE (*p* = 0.009), CMS (*p* = 0.031), and SST (0.026) were positively correlated to external rotation, while VAS (*p* = 0.020) was negatively related. Abduction peaks were positively related to CMS (*p* = 0.011) and SANE (*p* = 0.037), as well as abduction ROM (SANE, *p* = 0.031; CMS, *p* = 0.014).

## 1. Introduction

Reverse total shoulder arthroplasty (RTSA) is one of the most common surgical procedures performed in patients affected by several shoulder diseases, such as irreparable rotator cuff tears, rotator cuff arthritis, and fixed shoulder instability [1,2,3].

In the USA, the number of RTSAs performed annually increased by 191.3%, reaching 63,845 surgeries in 2017 [4]. In Italy, shoulder replacements’ incidence increased from 7.5 to 21.7 cases per 100,000 residents [5]. Moreover, RTSA volumes will continue to increase [4,5]. 

Lateralization and distalization of prosthetic implants were previously identified as two main influencing factors affecting shoulder biomechanics after surgery [6,7]. Lateralization and distalization have an impact on the position of the new center of rotation of the joint, the forces at the bone–implant interface, the implant stability, and the deltoid’s lever arm and pre-tensioning [6,7]. The overall lateralization and distalization are due to the design and size of the components of the implant, their positioning, and the surgical technique [7,8]. A surgical technique used to increase lateralization is the BIO-RSA, which consists of placing a bone graft between the baseplate and the glenoid [6,7,9].

Nowadays, preoperative 3D planning allows the surgeon to use a 3D model of the patient’s scapula, created through a preoperative CT scan, to test different sizes and positions of the implant’s components [10,11,12]. The preoperative plan is then uploaded to computer-assisted intraoperative navigation software that guides the surgeon in positioning the implant [10,11,12]. These software techniques can increase accuracy and precision in the baseplate and screw positioning and influence surgical choices; however, the decision-making process is still based on the surgeon’s experience [10,11,12,13].

Previous studies investigated how different radiographic measures of lateralization and distalization could be related to clinical and kinematic outcomes after RTSA [7,14,15,16,17,18]. Lateralization shoulder angle (LSA) and distalization shoulder angle (DSA) were shown to be easily available and reproducible measures that provide an estimation of the overall lateralization and distalization with respect to anatomical reference points [14,15,16]. Higher LSA values were found to be related to increased internal/external rotation and forward elevation [14,15,16]. In addition, higher DSA values were found to be associated with higher forward elevation [15]. Moreover, from previous correlation analyses, LSA was found to be related to the Constant Murley Score (CMS), Simple Shoulder Test (SST), and Single Assessment Numeric Evaluation (SANE), while DSA was never correlated to clinical scales [14,15].

The objective of this study was to figure out how LSA and DSA are related to shoulder kinematics and clinical outcomes after RTSA. Our hypothesis was that LSA would correlate with higher internal–external rotation and shoulder elevation, while DSA would be associated with higher shoulder elevation. Moreover, the relationship between shoulder kinematics and clinical outcomes after RTSA was evaluated.

## 2. Materials and Methods

### 2.1. Study Design

In this study, 33 patients undergoing RTSAs between 2021 and 2022 were enrolled. The study group consisted of 19 females (57.58%) and 14 males (42.42%) with a mean age at surgery of 73 years (range from 50 to 85 years) and a mean follow-up of 14 months (range from 6 months to 2 years). Patients’ BMI ranged from 18.75 to 38.67, with a mean value of 27.29. Inclusion criteria were age between 50 and 85 years, no alteration of mental state, ability to return to the hospital for medical evaluation (clinical and radiologic), radiographic diagnosis of rotator cuff tear arthropathy, massive irreparable rotator cuff tear, and primary glenohumeral osteoarthritis. Exclusion criteria were neuromotor disorders, diagnosis of rheumatoid arthritis or inflammatory arthritis, fracture or necrosis of the humeral head, fracture sequelae of the humeral head, presence of cancer in the treated area, revision of a previous shoulder prosthetic implant, and less than six months of follow-up. All subjects approved and signed informed consent forms before participating in this study. Approval for this study was obtained from the local Ethical Committee (protocol 15.21 (OSS)).

### 2.2. Prosthetic Implant Design and Surgical Procedure

All procedures were performed via a deltopectoral approach using an Aequalis Tornier reverse-type prosthesis (Wright Medical Group Inc., Memphis, TN, USA) with the BIO-RSA technique. The prosthetic design was the Aequalis Reverse II on the glenoid side, and the Aequalis Ascend™ Flex on the humeral side. For all patients, the glenosphere’s tilt was 0°. The overall humeral neck shaft angle (NSA) was 145°, given by the sum of the humeral stem angle (132.5°) and the insert angle (12.5°). The glenosphere’s diameter and eccentricity and humeral retroversion are available in Table 1.

### 2.3. Radiographic Evaluation

**Figure 1:**
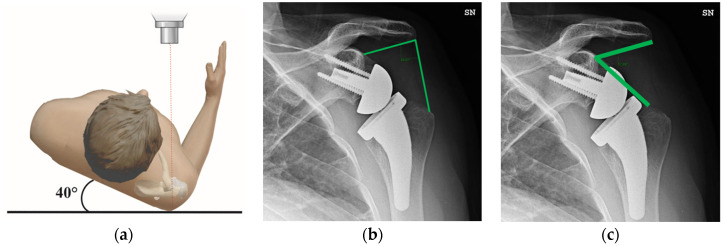
(**a**) True anteroposterior Grashey view radiography. This requires placing the patient in front of the X-ray beam with the glenoid rim tangential to them, so the shoulder is placed in an ipsilateral posterior oblique position. Radiographic indexes measured on anteroposterior Grashey view; (**b**) lateralization shoulder angle (LSA); (**c**) distalization shoulder angle (DSA).

LSA and DSA were measured on true anteroposterior Grashey view radiographs collected at the postoperative follow-up (Figure 1a) [19]. LSA is the angle formed by a line connecting the superior glenoid tubercle and the most lateral border of the acromion and a line connecting the latter point and the most lateral border of the greater tuberosity (Figure 1b) [14]. DSA is the angle formed by a line connecting the most lateral border of the acromion and the superior glenoid tubercle and a line connecting the latter point and the most superior border of the greater tuberosity (Figure 1c) [14]. Measurements on radiographic images were made by two independent clinicians (G.P., M.E.C.) to assess the inter-rater reliability.

### 2.4. Clinical and Kinematic Data

For clinical evaluation, the CMS, SST, and SANE scales were administered by the same clinician (M.E.C.) [20,21]. For pain investigation, the Visual Analogue Scale (VAS) (range: 0–10) was used [20].

The kinematic evaluation was performed using the Qualisys™ stereophotogrammetric system (Qualisys AB, Gothenburg, Sweden). Ten Miqus M3 cameras (sampling frequency, 100 Hz) and two Miqus Videos (sampling frequency, 25 Hz) were used to acquire markers’ (diameter, 8 mm) trajectories during the execution of tasks.

**Figure 2:**
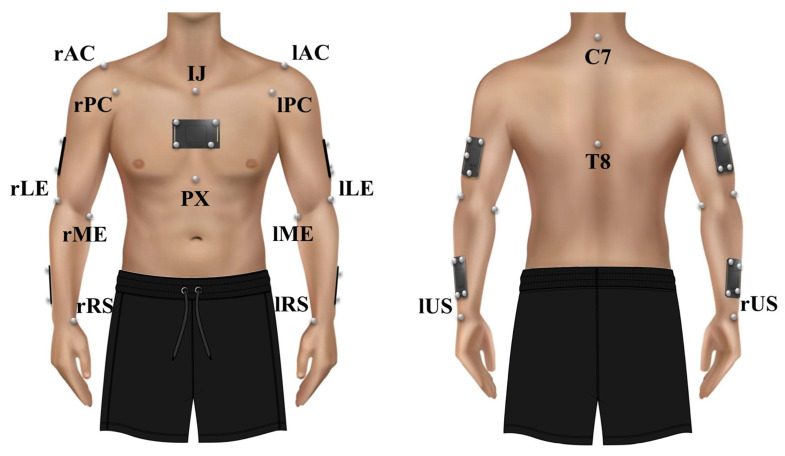
Anatomical markers placed following ISB recommendations: incisura jugularis (IJ); processus xiphoideus (PX); acromioclavicular joint (left/right) (AC); processus coracoideus (left/right) (PC); medial epicondyle (left/right) (ME); lateral epicondyle (left/right) (LE); radial styloid (left/right) (RS); ulnar styloid (left/right) (US); processus spinosus C7 (C7); processus spinosus T8 (T8). Rectangular-shaped clusters of markers placed on the thorax, upper arms, and forearms (bilaterally).

Sixteen markers were placed on anatomical landmarks through a palpation procedure performed by the same researcher (A.C.), following the ISB recommendations (Figure 2) [22]. Five rectangular-shaped clusters of four markers were placed on the thorax, humeri, and forearms for dynamic tracking (Figure 2) [23].

Markers’ trajectories were acquired and pre-processed in Qualisys Track Manager (QTM) software (v2022.8.5.0, Qualisys AB, Gothenburg, Sweden). Then, the 3D markers’ positions were imported into Visual 3D (C-Motion, Inc., Germantown, MD, USA) for kinematic model definition and kinematic analysis through a custom pipeline. Anatomical markers were used to determine the local coordinate system and orientation of the thorax and humeri [22].

The motion protocol included one static trial and five clinically relevant movements. The static trial consisted of maintaining the N-pose for 3 s, with arms down at the sides and palms facing inward. The dynamic tasks included elevations in the sagittal (Task 1), scapular (Task 2), and frontal (Task 3) planes, and two functional tasks, namely hand-to-nape (Task 4) and hand-to-back (Task 5). Patients were asked to perform five repetitions of each movement at a self-selected speed, up to the maximum possible ROM, without any pain condition. All five movements were performed bilaterally to avoid any undesired compensatory trunk movement. Only data from the affected side were further analyzed. The humerus orientation was expressed relative to the thorax. The rotation sequences used to evaluate humerothoracic (HT) angles were flexion–extension (FE), abduction–adduction (AA), and internal–external rotation (IER) (XYZ sequence in Visual 3D) for movements in the sagittal plane (Task 1) and AA, FE, and IER (YXZ sequence in Visual 3D) for movements in the frontal and scapular planes (Task 2 to Task 5) [24,25]. For all patients, the three central repetitions in the sagittal (FE), scapular (SCAP), and frontal (ABD) planes were selected for Task 1, Task 2, and Task 3, respectively. Similarly, the three central repetitions of the external rotation (ER) and abduction (ABD_HN) and internal rotation (IR) and abduction (ABD_HB) were selected for Task 4 and Task 5, respectively. For all tasks, the peak angles and ROMs used for subsequential analysis were calculated as the mean of the peak values and ROMs of the three central repetitions. ROM was defined as the difference between the maximum peak and the previous minimum.

### 2.5. Statistical Analysis

An inter-rater reliability analysis was conducted for the LSA and DSA indexes using the interclass correlation coefficient (ICC), with a 95% confidence interval. A mean rating (k = 2), absolute agreement, 2-way random ICC model was used. ICC values lower than 0.5 correspond to poor reliability; values between 0.5 and 0.75 correspond to moderate reliability; values between 0.75 and 0.9 correspond to good reliability; values greater than 0.9 correspond to excellent reliability [26].

A stepwise forward linear regression analysis between clinical or kinematic outcome measures and radiographic measurements was conducted. A correlation analysis was also conducted between clinical scores and HT peak angles. Pearson’s correlation was used in the case of normality of both distributions, and Spearman’s correlation was used otherwise [27,28]. The normality of the distributions was evaluated through the Shapiro–Wilk test. The statistical analyses were conducted using the software SPSS statistics v26 (IBM, SPSS, Inc., Chicago, IL, USA).

All analyses were performed by setting a level of statistical significance of *p* < 0.05 and a confidence interval of 95%.

## 3. Results

The inter-rater reliability analysis resulted in a good to excellent reliability for the LSA index (ICC mean: 0.93, range: 0.86–0.97) and an excellent reliability for the DSA index (ICC mean: 0.97, range: 0.95–0.99). Overall, the functional results—reported in terms of peak values—showed FE equals 143.3° ± 18.1° during elevation in the sagittal plane (Task 1), SCAP equals 131.4° ± 17.0° during elevation in the scapular plane (Task 2), ABD equals 132.2° ± 23.9° during elevation in the frontal plane (Task 3), ER equals 69.6° ± 20.9° and ABD_HN equals 128.1° ± 17.0° during the hand-to-nape task (Task 4), and IR equals −40.1° ± 17.8° and ABD_HB equals 37.4° ± 10.7° during the hand-to-back task (Task 5).

**Figure 3:**
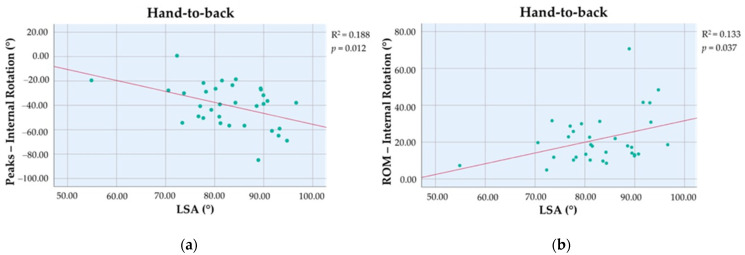
Stepwise forward linear regression analysis between lateralization shoulder angle (LSA) as independent variable and (**a**) peaks and (R^2^ = 0.188, *p* = 0.012) (**b**) range of motion (ROM) (R^2^ = 0.133, *p* = 0.037) for internal rotation during the hand-to-back task.

Greater LSA values were associated with higher peaks of IR, evaluated as negative values (β_unstandardized_ = −0.90, 95% CI: −1.58 to −0.21, *p* = 0.012, R^2^ = 0.188) and higher IR ROM (β_unstandardized_ = 0.58, 95% CI: −0.04 to 1.13, *p* = 0.037, R^2^ = 0.133) (Figure 3). LSA and DSA were not associated with other kinematic variables and clinical scores. Indeed, the two indexes were not considered statistically significative variables and were not included in the fitting model during the forward selection process.

From the Shapiro–Wilk test, all clinical scales were found to be not normally distributed, so Spearman’s correlation analysis was carried out to evaluate the correlation between clinical scores and kinematic outcomes. ER peak angles showed a significant negative relation with VAS (r = −0.404, *p* = 0.020) and a positive one with all the other clinical scales (SANE: r = 0.449, *p* = 0.009; CMS: r = 0.376, *p* = 0.031; SST: r = 0.388, *p* = 0.026) (Table 2). ABD peak angles were significantly related to SANE (r = 0.365, *p* = 0.037) and CMS (r = 0.435, *p* = 0.011) (Table 2). Also, ABD ROM showed a positive correlation to both SANE (r = 0.377, *p* = 0.031) and CMS (r = 0.426, *p* = 0.014) (Table 3). There was no other significant correlation between kinematics and clinical outcomes.

## 4. Discussion

Identifying easily available and reproducible indexes of lateralization and distalization related to RTSA outcomes could provide surgeons with guidelines about optimal implant positioning.

The results of this study showed that LSA (ICC mean: 0.93, range: 0.86–0.97) and DSA (ICC mean: 0.97, range: 0.95–0.99) have good to excellent inter-rater reliability. In previous studies, LSA and DSA showed moderate–good to excellent reliability, with an ICC mean value from 0.78 to 0.84 for LSA and from 0.66 to 0.81 for DSA [14,15]. Although LSA and DSA showed acceptable reproducibility as indexes derived from radiographic measurements, care must be taken in the interpretation of the results. Indeed, these angular measurements estimate humeral lateralization and distalization after RTSA with respect to the acromion and glenoid. Moreover, the LSA and DSA are correlated with each other; indeed, a lower LSA, i.e., more medial placement of the prosthetic implant, corresponds to a greater DSA, i.e., greater distance between the humerus and acromion [14].

In accordance with our hypothesis, LSA values were positively associated with IR. Indeed, higher values of LSA led to greater IR peak values (β_unstandardized_ = −0.90, *p* = 0.012, R^2^ = 0.188) and ROMs (β_unstandardized_ = 0.58, *p* = 0.037, R^2^ = 0.133) after RTSA. Erickson et al. also found a positive relationship between LSA and IR, showing IR increase with a greater LSA (*p* = 0.007) [16]. In addition, Erickson et al. showed increased IR corresponding to greater lateralization, also considering other measures besides LSA, namely the distance between the acromion and the glenosphere (*p* = 0.005) and the distance between the acromion and the greater tuberosity (*p* = 0.021) [16]. In contrast, Boutsiadis et al. found no statistically significant linear regression between LSA and shoulder IR (R^2^ = 0.010, *p* = 0.490) [14]. These differences in results could be explained by existing differences in surgical procedures. Indeed, restoring IR after RTSA could depend on subscapularis repair and postoperative tendon quality [29,30]. With an intact subscapularis, lateralization could increase the moment arm of the rotator cuff muscles and, consequently, IR [31]. In Erickson et al.’s and Boutsiadis et al.’s studies, part of the cohort had their subscapularis repaired [14,16]. In our study, the subscapularis tendon was repaired in all the patients.

In contrast with our hypothesis, LSA and DSA did not show any association with shoulder elevation. Similar to previous studies, no statistically significant linear regression was found between LSA and active shoulder abduction [14,15]. This could be due to the glenosphere’s eccentric positioning, which could decrease the effect of lateralization on abduction [32]. According to our study, Boutsiadis et al. found no statistically significant linear regression between active abduction and LSA (R^2^ = 0.04, *p* = 0.28) or DSA (R^2^ = 0.09, *p* = 0.45) [14]. Similarly, Berthold et al. found no significant correlations between active abduction and LSA (r = 0.030, *p* = 0.824) or DSA (r = 0.145, *p* = 0.283) [15]. In contrast to our study, Boutsiadis et al. found a positive linear regression between LSA and postoperative forward elevation (R^2^ = 0.2, *p* = 0.008) and an inverse linear regression between DSA and forward elevation (R^2^ = 0.2, 0 = 0.004) [14]. Berthold et al. found a significant correlation between final forward elevation and LSA (r = −0.276, *p* = 0.033) and DSA (r = 0.299, *p* = 0.02) [15]. These differences in results could be due to differences in the characteristics of the implants, patients’ demographics, sample size, follow-up period, and acquisition protocol. In the mentioned studies, the movement protocol and the HT angle measurement methods were not always specified or were measured using a goniometer, and IR was measured as the reached spinal level [7,8,14,15,16,33]. In the current study, HT angles were analyzed through a stereophotogrammetric system, which allows objective and accurate measurement [34]. The high heterogeneity among the studies in the literature makes direct comparisons that could lead to unambiguous conclusions difficult. However, refining diagnostic techniques, radiographic measurements, clinical–functional assessments, and further investigating the relationship between prosthetic implant placement and clinical outcomes may have important implications in clinical practice. Indeed, elucidating the effect of distalization or lateralization on clinical outcomes in advance could positively influence clinicians’ decision making in the management of patients requiring RTSA, making treatment more patient-oriented according to a personalized care approach.

From the correlation analysis, CMS was positively correlated to ER (r = 0.376, *p* = 0.031), ABD peak values (r = 0.435, *p* = 0.011), and ROM (r = 0.426, *p* = 0.014). Indeed, the CMS questionnaire includes direct questions about ability in terms of execution of activities of daily living (ADLs) and ROM [20]. Also, the SANE score was related to ER (r = 0.449, *p* = 0.009), ABD peak values (r = 0.365, *p* = 0.037), and ROM (r = 0.377, *p* = 0.031). This is in accordance with previous studies, where it was shown to be related to more extensive clinical scales such as the American Shoulder and Elbow Surgeons (ASES) and CMS [21]. SST related only to ER peak values. SST is a score based on 12 “yes” or “no” questions; therefore, it likely could not discriminate different stages of the postoperative RTSA condition [20]. Moreover, SST is more susceptible to postoperative ceiling effects [35]. In our study, patients with a limited ER reported higher pain values (r = −0.404, *p* = 0.020). This may be explained because of changes in deltoid function and pathologies that affect rotator cuff muscles, which make the ER one of the less repaired movements after RTSA [36].

Our study was retrospective with a small sample size, evaluated at heterogeneous follow-ups ranging from a minimum of six months to a maximum of two years after surgery. Moreover, our study did not consider postoperative rehabilitation programs nor clinical–functional and structural assessment before surgery. Interpretation of the results must also consider the inherent influence of soft-tissue artifacts in assessing shoulder kinematics using a stereophotogrammetric system with passive photo-reflective markers. In the present study, radiographic measures of LSA and DSA did not take into account scapular tilting or the degree of humerus rotation. Future studies should include a larger cohort to allow the inclusion of more influencing factors in the analysis. Indeed, implant characteristics such as the glenosphere’s diameter, eccentricity, tilting, humerus rotation, and scapular orientation are parameters that could influence the impingement-free motion after surgery in internal and external rotation [7,37,38]. Moreover, comorbidity, shoulder pathology, and patients’ demographics could also be influencing factors for clinical and kinematic outcomes [39,40,41]. Furthermore, in future studies, patients with the same follow-up of at least two years will be enrolled. Indeed, internal rotation and external rotation are the two movements typically more affected by RTSA [7,31]. Kim et al. showed that more than two years after surgery, IR and ER almost reached their maximum recovery [36]. For this reason, a large cohort of patients undergoing RTSA will be evaluated at the same long-term follow-up to report clinically relevant results considering multiplanar functional movements requiring significant shoulder IR and ER to be correctly executed, as typical movements of ADLs.

## 5. Conclusions

In conclusion, our study pointed out that positioning the prosthetic implant with higher LSA values could lead to higher IR peak values and IR ROM after RTSA. However, the association of LSA and DSA with other kinematic and clinical outcomes was not statistically significant. Further studies are needed to assess the clinical significance of these results because of the small sample available for this analysis and the great number of variables that could influence RTSA outcomes.

## Figures and Tables

**Table 1:** Surgical technique and implant characteristic variables.

Subject	Humeral Retroversion (°)	Eccentricity (mm)	Glenosphere Diameter (mm)	LSA (°)	DSA (°)
1	10	3.5	39	93.03	48.96
2	10	1.5	36	82.98	62.46
3	10	1.5	36	80.94	56.62
4	20	1.5	36	89.94	57.79
5	10	3.5	39	91.68	34.46
6	10	1.5	36	81.06	54.10
7	30	3.5	39	89.97	53.14
8	10	1.5	36	81.18	53.38
9	10	1.5	36	76.70	59.10
10	20	3.5	36	84.23	57.84
11	20	3.5	39	72.32	70.66
12	10	1.5	36	86.07	52.65
13	10	3.5	39	94.75	35.80
14	10	3.5	42	93.24	45.71
15	20	1.5	36	80.18	51.68
16	10	1.5	36	83.62	59.01
17	10	3.5	36	78.23	51.48
18	20	1.5	36	90.76	41.25
19	10	1.5	36	88.56	59.00
20	10	3.5	39	54.87	102.37
21	10	3.5	39	79.31	60.19
22	10	1.5	36	89.44	47.43
23	10	1.5	36	96.62	31.15
24	10	1.5	36	81.49	57.34
25	20	3.5	36	70.56	72.10
26	10	1.5	36	73.74	63.43
27	10	3.5	42	89.38	45.72
28	10	3.5	39	84.32	55.95
29	10	3.5	39	77.68	63.40
30	10	3.5	42	77.71	55.02
31	10	1.5	36	77.04	64.03
32	20	1.5	36	88.90	54.97
33	10	1.5	36	73.40	72.62

LSA: lateralization shoulder angle; DSA: distalization shoulder angle.

**Table 2:** Spearman’s correlation analysis between clinical outcomes and peak angles.

Peaks	VAS		SANE		CMS		SST	
	r	*p*	r	*p*	r	*p*	r	*p*
FE	0.138	0.444	0.105	0.561	0.083	0.645	−0.009	0.962
SCAP	−0.013	0.943	0.132	0.463	0.022	0.901	−0.045	0.805
ABD	−0.026	0.885	0.365	0.037 *	0.435	0.011 *	0.320	0.070
ABD_HN	−0.233	0.193	0.018	0.920	0.046	0.798	0.009	0.961
ER	−0.404	0.020 *	0.449	0.009 *	0.376	0.031 *	0.388	0.026 *
ABD_HB	0.151	0.401	0.202	0.912	0.209	0.244	0.165	0.358
IR	−0.237	0.184	−0.066	0.714	−0.129	0.473	−0.067	0.710

* Statistically significant correlation (*p* < 0.05). VAS: Visual Analogue Scale; SANE: Single Assessment Numeric Evaluation; CMS: Constant Murley Score; SST: Simple Shoulder Test; FE: flexion–extension, elevation in the sagittal plane; SCAP: scaption, elevation in the scapular plane; ABD: abduction, elevation in the frontal plane; ABD_HN: abduction during hand-to-nape task; ER: external rotation during hand-to-nape task; ABD_HB: abduction during hand-to-back task; IR: internal rotation during hand-to-back task.

**Table 3:** Spearman’s correlation analysis between clinical outcomes and ROMs.

ROMs	VAS		SANE		CMS		SST	
	r	*p*	r	*p*	r	*p*	r	*p*
FE	0.099	0.582	0.121	0.503	0.043	0.813	−0.012	0.948
SCAP	−0.078	0.644	0.261	0.142	0.015	0.934	0.017	0.927
ABD	−0.057	0.753	0.377	0.031 *	0.426	0.014 *	0.293	0.098
ABD_HN	−0.225	0.209	0.106	0.556	0.027	0.883	0.023	0.889
ER	−0.156	0.385	0.239	0.180	0.194	0.280	0.165	0.359
ABD_HB	0.040	0.827	0.134	0.459	0.158	0.381	0.191	0.287
IR	0.198	0.269	0.257	0.150	0.173	0.335	0.182	0.311

* Statistically significant correlation (*p* < 0.05). VAS: Visual Analogue Scale; SANE: Single Assessment Numeric Evaluation; CMS: Constant Murley Score; SST: Simple Shoulder Test; FE: flexion–extension, elevation in the sagittal plane; SCAP: scaption, elevation in the scapular plane; ABD: abduction, elevation in the frontal plane; ABD_HN: abduction during hand-to-nape task; ER: external rotation during hand-to-nape task; ABD_HB: abduction during hand-to-back task; IR: internal rotation during hand-to-back task.

## Data Availability

Data are available from the corresponding author on reasonable request.
